# Effects of Di-(2-Ethylhexyl) Phthalate (DEHP) on Gamete Quality Parameters of Male Koi Carp (*Cyprinus carpio*)

**DOI:** 10.3390/cimb45090467

**Published:** 2023-09-11

**Authors:** Kampan Bisai, Vikash Kumar, Arpita Roy, Satya Narayan Parida, Souvik Dhar, Basanta Kumar Das, Bijay Kumar Behera, Manoj Kumar Pati

**Affiliations:** 1 Biotechnology Laboratory, ICAR-Central Inland Fisheries Research Institute, Barrackpore, Kolkata 700120, West Bengal, India; kampanbisai@gmail.com (K.B.);; 2 Department of Fishery Sciences, Vidyasagar University, Midnapore, Paschim Medinipur 721102, West Bengal, India; 3 College of Fisheries, Rani Lakshmi Bai Central Agricultural University, Gwalior Road, Jhansi 284003, Uttar Pradesh, India

**Keywords:** di-(2-ethylhexyl) phthalate (DEHP), koi carp (*Cyprinus carpio*), gamete quality, sex steroids, gene expression

## Abstract

In this study, we evaluated gamete quality parameters of mature male koi carp (*Cyprinus carpio*) exposed to three different concentrations (1, 10, and 100 µg/L) of di-(2-ethylhexyl) phthalate (DEHP). After 60 days of exposure, there was a significant decrease in the gonadosomatic index (GSI) of males exposed to 10 and 100 µg/L of DEHP. Histological analysis of the testes revealed impaired histoarchitecture, including inflammatory cells, intratubular vacuoles, and swollen seminiferous tubules in treatment groups. Gamete quality parameters like sperm production, motility, spermatocrit, and sperm density values were significantly decreased at the 10 and 100 µg/L concentrations. Biochemical compositions, including glucose, cholesterol, and total protein levels, were significantly changed in the treatment groups. Similarly, the ionic compositions of seminal fluid (Na, K, Ca, and Mg) also varied in the treatment groups. Furthermore, the 11-ketotestosterone levels were decreased, and the 17-β estradiol levels were increased in the DEHP-treated groups. The mRNA expression levels of reproduction-related genes, including Fshr, Lhr, Ar, Erα, and Erβ, were significantly changed in the DEHP-treated males in a dose-dependent manner. In conclusion, the findings of this study confirmed that environmentally relevant exposure to DEHP may contribute to a decline in the gamete quality of male fishes.

## 1. Introduction

Male infertility has increased globally over the past few years [1]. Declining semen quality is a major contributor to the increased prevalence of male infertility. Research suggests that male fertility can be affected by any factor that affects the quality of semen [2]. Environmental pollutants play a significant role in the decline in sperm quality, although the exact mechanisms of action are not clearly understood [3,4,5]. Pollutants can interfere with the action of hormones via multiple mechanisms and produce inflammatory problems in the reproductive system, possibly leading to male infertility.

Di-(2-ethylhexyl) phthalate (DEHP) is one of the most widely used manmade chemicals, which is also considered a ubiquitous environmental pollutant [6]. It is used as a nonreactive plasticizer not only in polyvinyl chloride (PVC) but also in a broad range of industrial processes and consumer products, including cosmetics, medical devices, building materials, automobile parts, and food packaging materials [7,8]. This substance is not chemically attached to plastics and commonly leaks out in liquid form from these items into the surrounding environment [9,10], affecting individuals who are in close contact [11]. As a result of its extensive application and production, more than 2 million tonnes are produced globally every year [12]. DEHP is measured at 1 µg/L in drinking water, 10 µg/L in surface water, and 98–218 µg/L in polluted areas [13,14]. Fish are exposed to DEHP present in the water column, sediments, and also via their diet [15]. DEHP can be hydrolyzed to its primary metabolite, mono-(2-ethylhexyl) phthalate (MEHP) [16].

In mammals, previous studies indicate that DEHP has the potential to induce a wide range of reproductive impairments, including the disruption of spermatogenesis, imbalanced steroid hormone levels, and infertility in males [17,18,19,20,21,22,23,24,25,26]. However, very few studies have revealed the effects of DEHP on the male reproductive systems of aquatic organisms such as fish. DEHP has been found to reduce the fertilization rate of male zebrafish (*Danio rerio*) when they were injected with 5000 mg/kg (body weight) of DEHP for 10 days [15] or exposed to 0.2 to 20 µg/L of DEHP for 21 days [27]. Marine medaka (*Oryzias melastigma*) dosed with 100 and 500 µg/L of DEHP from hatchling to maturity stage showed a reduction in male fertility as well [28]. Furthermore, exposure to 1–100 µg/L of DEHP for 30 days in mature goldfish (*Carassius auratus*) disrupted both pituitary and testicular hormonal functions and reduced sperm quality [29]. Various reproductive impairments, including damage to testes, the disruption of sex hormone production, and inhibited spermatogenesis, were observed when male zebrafish (*D. rerio*) were exposed to 10–100 µg/L of DEHP for 90 days [30]. The altered histoarchitecture of testes and changes in testosterone levels were observed in African catfish (*Clarias gariepinus*) exposed to DEHP (200 and 400 µg/L) for 14 days [31]. However, these data have been limited to studies involving laboratory animals, and in none of these investigations using fish models were clear associations found between DEHP and sperm motility, spermatocrit value, sperm density, or the biochemical composition of semen. Sperm production, motility, spermatocrit value, and sperm density are key determinants of male fertility in fish [32,33].

The main objective of the present study was to determine the effect of DEHP, at concentrations occurring in aquatic environments, on gamete quality parameters using mature male koi carp (*Cyprinus carpio*) as a model organism. We investigated a number of parameters, including the gonadosomatic index (GSI), testicular histopathology, semen volume as well as pH, sperm motility, spermatocrit value, sperm density, and biochemical parameters of semen after 60 days of exposure to DEHP at three different concentrations, i.e., 1, 10, and 100 µg/L. Exposure to DEHP may potentially impact molecular mechanisms in the reproductive processes of fishes. Thus, analyses of sex steroid levels and mRNA expression in several reproduction-related genes were also carried out in this study. We also confirmed the accumulation of DEHP in tissue samples of exposed fishes using GC-MS technology. Our study provides valuable information to help in assessing the potential impact of DEHP on aquatic life.

## 2. Materials and Methods

### 2.1. Chemicals Used

Technical-grade di-(2-ethylhexyl) phthalate (99% purity) and acetone (99% purity) were acquired from SRL Laboratories Ltd., Mumbai, Maharastra, India. DEHP was dissolved in acetone (100 g/L) to create the stock solution and kept at 4 °C until use. To produce the exposure concentrations, serial dilutions of the stock solution were performed as needed. Every additional chemical employed in this research was of analytical grade.

### 2.2. Experimental Fish

At the outdoor culture unit of the Department of Fishery Sciences, Vidyasagar University, Midnapore, West Bengal, India, mature male koi carps (*C. carpio*) collected from a local fish dealer with an average weight of 36.64 ± 0.86 g and an average length of 14.82 ± 0.62 cm were acclimatized for 20 days in a cement cistern of 3000 L capacity. They were divided randomly into different groups for the exposure experiment after acclimatization.

### 2.3. Exposure Experiment

Four different groups (one control and three DEHP-treated) were made to find out the consequence of DEHP on the gamete quality parameters of male *C. carpio*. Twelve glass tanks (180 L) were used (three tanks for each group), and 12 adult male fish were kept in each tank. DEHP was supplied through water at three different concentrations in distinct experimental groups. One group was treated with a lower concentration of DEHP at 1 µg/L of water, another with a medium concentration of 10 µg/L of water, and a higher concentration treated group with 100 µg/L of water. Similarly, for comparison, another group was kept as a control (without DEHP). The solutions for all groups, including the control group, contained 0.001% (*v*/*v*) acetone as a solvent. The experiment lasted 60 days, and during this time, the fishes were fed commercial carp feed (CP Aquaculture Pvt. Ltd., Chennai, Tamilnadu, India) twice daily at 4% body weight. At weekly intervals, all the water in each experimental tank was replaced, and various doses of DEHP were prepared from stock solution, which the fishes were exposed to. The water temperature ranged from 30 °C to 32 °C during the experimental trial, and other water quality parameters, including the pH, dissolved oxygen, and alkalinity of the water, were 7.2 ± 0.2, 5.8 ± 0.4 mg/L, and 82 ± 1.2 mg/L, respectively. The experiment was conducted under aerated conditions with a 12 h light and 12 h dark photoperiod.

### 2.4. GSI and Histopathological Study

After the exposure of 60 days, fish from all groups were sacrificed by being anaesthetized with tricaine methanesulfonate (MS-222) followed by a big blow to the head, and testes were collected and weighed to calculate the GSI. GSI is defined as the testes weight (g) to the total fish weight (g) × 100. After that, pieces of the testes were used for histopathological studies as well as the quantification of DEHP. After being fixed in Bouin’s solution for 24 h, paraffin-embedded tissues were sectioned at a thickness of 5 µm using a rotary microtome (RM 2025, Leica Biosystems, Nußloch, Baden-Wurttemberg, Germany). Hematoxylin and Eosin stain (H & E stain) was used to stain the sections, and a light microscope (Axio Lab A1, Carl Zeiss, Oberkochen, Baden-Wurttemberg, Germany) was used to analyze the histoarchitecture of the testes. 

### 2.5. Quantification of DEHP

The concentrations of DEHP were determined from the pooled samples of liver and testes of each experimental group via a gas chromatography mass spectrometer (ISQ 7000 GC-MS, Thermo Scientific, Waltham, MA, USA) equipped with a 30 m × 0.25 mm × 0.25 µm capillary column (TG-35MS Column, Thermo Scientific, Waltham, MA, USA) in electron impact and selective ion monitoring (SIM) mode. The tissue sample processing and conditions of GC-MS are briefly described in the Supplementary file (Section S1).

### 2.6. Collection of Semen

Fishes were collected and dried from the control and DEHP-treated groups. Each male was only stripped once, and the complete amount of expressible semen was individually collected by gently pressing their bilateral abdomens. The semen was put into a dry and clean glass tube. Semen was collected with care to prevent it from becoming contaminated with water, urine, blood, or feces. The tubes were immediately capped and brought on ice to the laboratory for analysis.

### 2.7. Evaluation of Semen Volume and pH

Semen samples of the control and DEHP-treated fishes were collected separately into 10 mL measuring glass tubes (Borosil Glass Works Ltd., Mumbai, Maharastra, India), and the volume was recorded. The pH of sperm was measured using a standard pH meter (A211 Benchtop pH Meter, Thermo Scientific, Waltham, MA, USA) within 30 min of sampling.

### 2.8. Evaluation of Sperm Motility

Within the first hour of semen collection, the sperm motility in each sample was assessed. A total of 10 µL of semen was pipetted onto a microscope slide coated with 1% bovine serum albumin (BSA). To activate the sperm, 25 µL of 0.3% NaCl solution was added. The motility levels of sperm from the control and DEHP-treated fishes were examined using a dark field microscope (Axio Scope A1, Carl Zeiss, Oberkochen, Baden-Wurttemberg, Germany). Spermatozoa were only considered mobile while moving forward. However, those that were only vibrating were considered immobile. The percentage of motility was arbitrarily chosen on a scale of 0 to 10, with 0 representing 0% motility and 10 denoting 100% motility. The time between the last spermatozoa’s activation and complete cessation of activity in that field was used to calculate the duration of the motility. Three repetitions of each sample were used to observe sperm motility. To reduce the level of variation, all the sperm motility observations were performed by a single person.

### 2.9. Evaluation of Spermatocrit Value

For the measurement of spermatocrit value, semen was placed inside hemoseal wax-sealed microcapillary tubes with dimensions of 75 mm in length, 1 mm in inner diameter, and 0.1 mL capacity (Top Tech Lab Equipment’s Pvt. Ltd., Mumbai, Maharashtra, India). A meter scale in mm was used to measure the volume (length) of semen in capillaries. Later, the capillary tubes were centrifuged at 1500× *g* for 10 min. The volume of white packed cells as a percentage of the total volume of semen is known as spermatocrit. Within one hour following the collection of the semen, the spermatocrit of each sample was determined in triplicate.

### 2.10. Evaluation of Sperm Density

To assay the sperm density of the control and DEHP-treated fishes, at first, Hayem’s solution (5 g of Na_2_SO_4_, 1 g of NaCl, 0.5 g of HgCl_2_, and 200 mL of double distilled water) was used to dilute the semen samples at a ratio of 1:1000. Then, using light microscopy (Axio Lab A1, Carl Zeiss, Oberkochen, Baden-Wurttemberg, Germany), a droplet of the diluted semen was applied on a hemocytometer slide (0.1 mm depth, Marienfeld Superior, Lauda-königshofen, Baden-Wurttemberg, Germany) with a coverslip. The hemocytometer slide was kept in a wet environment for at least 10 min before cell counting to allow sperm sedimentation. According to Alavi et al. [34], the number of spermatozoa was then counted in triplicate for each sample.

### 2.11. Biochemical Analysis of Seminal Fluid

Centrifugation at 10,000× *g* was applied to separate the seminal fluid from the semen for 10 min at 4 °C. To prevent spermatozoa contamination, seminal fluid was centrifuged twice and kept in a microcentrifuge tube (Tarsons Products Pvt. Ltd., Kolkata, West Bengal, India) at –20 °C until analysis began.

The glucose and cholesterol levels of the seminal fluid were measured individually using Spinreact kits purchased from ARK Diagnostics Ltd., Mumbai, Maharastra, India. However, the total protein in the seminal fluid was measured by using the Anamol Total Protein kit purchased from Anamol Laboratories Pvt. Ltd., Thane, Maharastra, India. All samples were treated and analyzed in triplicate via UV-VIS spectrophotometry (UV 3600i Plus, Shimadzu, Kyoto-Shi, Kyoto, Japan) following the manufacturer’s protocol.

The concentrations of sodium (Na), potassium (K), calcium (Ca), and magnesium (Mg) in the seminal fluid were determined using the procedure previously described by Rosengrave et al. [35] using atomic absorption spectroscopy (AA-7000, Shimadzu, Kyoto-Shi, Kyoto, Japan) with an air–acetylene flame. The standard solutions of Na, K, Ca, and Mg were purchased (Sigma-Aldrich, Merck Group, St. Louis, MO, USA), and the solutions were diluted as necessary to obtain working standards. To measure Na and K, 25 μL samples were diluted 200 times with demineralized water and measured at 589.6 nm and 766.5 nm, respectively. During the analysis of Na, a burner angle of 45° was adjusted to reduce the sensitivity. Samples and combined standards of Na (1.2 mmol/L) and K (0.04 mmol/L) contained 1 g/L of Cs (chloride) for ionization suppression. However, for Ca and Mg, 100 μL samples were diluted 50 times with demineralized water and measured at 422.7 nm and 285.2 nm, respectively. For the background correction of Mg, a deuterium continuum lamp was employed. Samples and the combined standard of Ca (0.1 mmol/L) and Mg (0.02 mmol/L) contained 2.5 g/L of La (chloride) as a releasing agent. During the analysis of Ca, 1 g/L of HNO_3_ was used for the complete recovery of Ca. Measurements of Na, K, Ca, and Mg were carried out in triplicate for each sample.

### 2.12. Determination of Sex Steroids

Blood samples were collected from the caudal peduncle of fishes from the DEHP-treated groups and the control group using a syringe and were placed in a 2 mL centrifuged tube without any anticoagulant and kept for 20–25 min at room temperature. After that, clotted blood samples were centrifuged at 6000× *g* for 8 min in cold conditions (4 °C), and serum was collected and stored at –20 °C for further use.

17-β estradiol and 11-keto testosterone levels were analyzed using a commercial enzyme-linked immunosorbent assay (ELISA) kit purchased from Krishgen Biosystems, Mumbai, Maharastra, India. To quantify the levels of hormone mentioned above in the serum, all samples were equilibrated at room temperature and mixed via gentle inversion before use. The assays were performed following the manufacturer’s protocol using a microplate absorbance reader (iMark 13863, Hercules, CA, USA). 

### 2.13. RNA Extraction and cDNA Synthesis

Following the manufacturer’s instructions, the Tri-reagent (Sigma-Aldrich, Merck Group, St. Louis, MO, USA) was used to extract total RNA from the fish testes in each experimental group. Briefly, a 100 mg tissue sample from each group was homogenized with 1 mL of Tri-reagent in ice-cold condition. Then, 200 µL of chloroform was added to each homogenate for complete lysis, and centrifugation (12,000× *g*) at 4 °C for 15 min was carried out for phase separation. The aqueous phase containing RNA was transferred to a new centrifuge tube. Then, 500 µL of isopropyl alcohol was mixed with the aqueous phase and incubated for 10 min at room temperature. For RNA precipitation, the mix of each sample was centrifuged (12,000× *g*) for 10 min at 4 °C. Precipitate RNA was washed with 75% chilled ethanol (1 mL) and centrifuged again (7500× *g*) for 5 min at 4 °C. Finally, each RNA pellet was air dried, and the required quantity of nuclease-free water was added. The RNA concentration of each sample was assessed using a nano-drop (Eppendorf AG 22331, Barkhausenweg, Hamburg, Germany), which was then subjected to DNAse I treatment (Turbo DNA free kit, Thermo Scientific, Waltham, MA, USA). The quality of each RNA sample was also evaluated on 1% agarose gel. Each RNA sample was used to synthesize cDNA using a First Strand cDNA synthesis kit following the manufacturer’s instructions (K1622, Thermo Scientific, Waltham, MA, USA). Briefly, 5 µg of RNA and 1 µL of random hexamer primer were mixed in a sterile tube and made to 12 µL with the addition of nuclease-free water and incubated at 65 °C for 5 min. Each mix was then cooled down on ice. After that, 4 µL of 5× reaction buffer, 1 µL of RNase inhibitor, 2 µL of 10 mM dNTP, and 1 µL of reverse transcriptase were added, and the total volume of each tube was found to be 20 µL. Finally, each tube was incubated for 5 min at 25 °C, followed by 60 min at 42 °C. The synthesized cDNA samples were stored at −20 °C until further use. The housekeeping gene, i.e., 18S rRNA, was used to amplify each cDNA sample. A primer set was designed for each gene and amplified (Figure S1) in Thermal Cycler Gene Amp PCR System 9700 (Applied Biosystems, Foster City, CA, USA) (briefly stated in Section S2 of the Supplementary file).

### 2.14. Quantitative Real-Time PCR

Using a real-time PCR system (Light Cycler 480, Forrenstrasse, Rotkreuz, Switzerland), quantitative PCR (qPCR) was performed. Primer sets used to amplify Fshr, Lhr, Ar, Erα, and Erβ are listed in Table 1. For sample normalization, the housekeeping gene 18S rRNA was employed. The overall reaction volume for each sample was 20 μL, containing 2 μL of cDNA as a template, 1 μL of forward primer (5 pmol), 1 μL of reverse primer (5 pmol), 10 μL of 2× SYBR green I mix, and 6 μL of nuclease-free water supplied within the kit. The qPCR program included a pre-incubation step at 95 °C, 40 cycles of amplification at 95 °C for 10 s, annealing at the appropriate temperature for each gene (58 °C for Fshr, 56 °C for Lhr and Ar, 55 °C for Erα, and 54 °C for Erβ) for 10 s and extension for 10 s at 72 °C. Melt curve analysis at 95 °C for 5 s, 65 °C for 1 min, and 97 °C for 1 min was used to confirm the specificity of qPCR. The samples were chilled for 10 s at 40 °C. Following the 2^−ΔΔCT^ approach, the data were represented as the fold-change in expression relative to the 18S rRNA gene.

### 2.15. Statistical Analysis

The formats for all data are mean ± standard error of the mean (SEM). One-way analysis of variance (ANOVA) followed by Tukey’s multiple comparison test performed with the SPSS statistical software package (version 25.0, IBM Corp., Armonk, NY, USA) was used to analyze the differences between the control and different exposure groups for GSI, accumulation of DEHP in tissues, semen volume as well as pH, sperm motility, spermatocrit value, sperm density, biochemical parameters of semen, steroid levels in serum, and relative gene expression. At *p* < 0.05, the significant levels were reported.

## 3. Results

### 3.1. Changes in GSI and Histopathology

**Figure 1:**
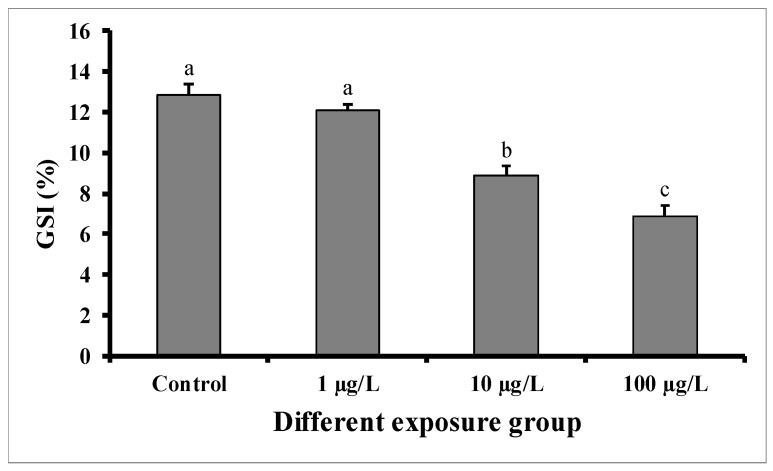
GSI (%) of male mature koi carp exposed to nominal 1, 10, and 100 µg/L of DEHP for 60 days. Data are expressed as mean ± SEM (*n* = 6), and values with different superscript letters (a denotes the highest value, followed by b and c) significantly differ at 0.05 level.

GSI significantly decreased in males exposed for 60 days to the medium (10 µg/L) and highest concentration (100 µg/L) of DEHP, but not in those exposed to the lower concentration (1 µg/L) in comparison with control males (Figure 1). In the control group, the GSI was 12.84 ± 0.54 (%), while in the group treated with 100 µg/L of DEHP, the value was found to be 6.86 ± 0.56 (%). The GSI of the group treated with 10 µg/L of DEHP was 8.88 ± 0.56 (%).

**Figure 2:**
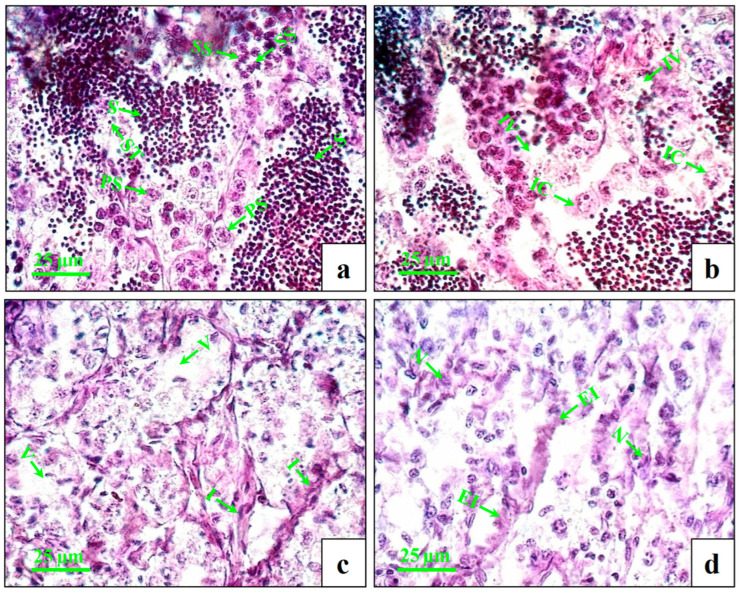
Histopathological changes in testes of male mature koi carp exposed to nominal 1, 10, and 100 µg/L of DEHP for 60 days. Control group (**a**) shows intact seminiferous tubules (ST) with primary spermatocyte (PS), secondary spermatocyte (SS), and spermatids (S). Group treated with 1 µg/L of DEHP (**b**) shows intratubular vacuoles (IV) and inflammatory cells (IC). Group treated with 10 µg/L of DEHP (**c**) shows vacuolization (V) and inflammation (I). Group treated with 100 µg/L of DEHP (**d**) shows enhanced inflammation (EI) and necrosis (N).

The testes of the control fish showed intact seminiferous tubules with primary and secondary spermatocytes and spermatids (Figure 2a). At lower concentrations (1 µg/L), very few numbers of inflammatory cells and intratubular vacuoles were observed (Figure 2b). At the medium concentration (10 µg/L), increasing numbers of inflammatory cells and intratubular vacuoles (Figure 2c) were observed. At higher concentrations (100 µg/L), the changes were more distinct with swollen seminiferous tubules, further enhancing the inflammation and necrosis (Figure 2d).

### 3.2. Assessment of DEHP in Tissue Samples

**Figure 3:**
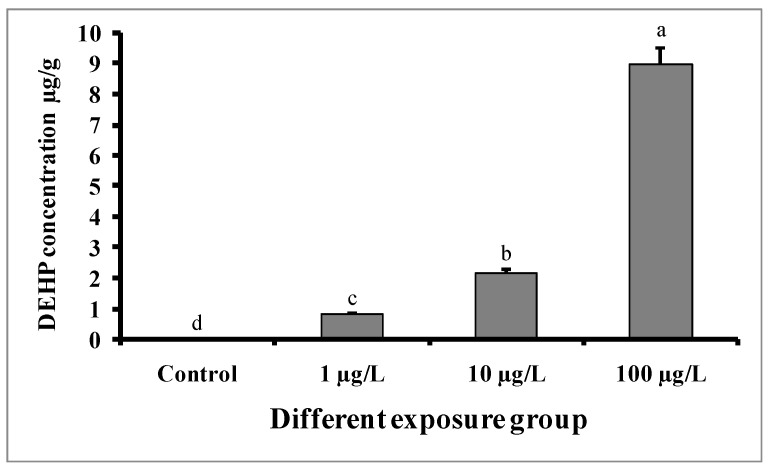
Accumulation of DEHP (µg/g) in pooled samples of liver and testes tissue of male mature koi carp exposed to nominal 1, 10, and 100 µg/L of DEHP for 60 days. Data are expressed as mean ± SEM (*n* = 6), and values with different superscript letters (a denotes the highest value, followed by b, c, and d) significantly differ at the 0.05 level.

Figure 3 shows that DEHP accumulated in the pooled liver and testes tissue samples of all experimental groups. Significantly higher DEHP was detected in fish treated with higher doses (100 µg/L) of DEHP, followed by those treated with medium (10 µg/L) and lower (1 µg/L) doses of DEHP. The concentration of DEHP in the tissue samples of the group treated with 100 µg/L of DEHP was 8.96 ± 0.52 µg/g. However, in the case of the control group, DEHP was below the limit of detection (LOD).

### 3.3. Changes in Semen Volume and pH

**Figure 4:**
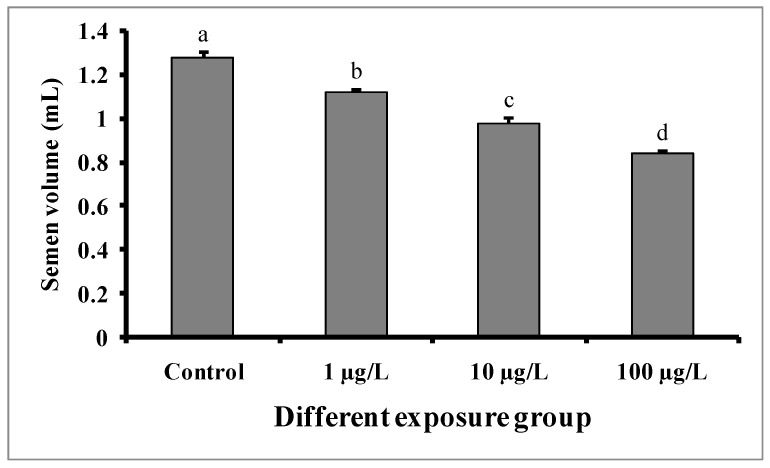
Semen volume (mL) of male mature koi carp exposed to nominal 1, 10, and 100 µg/L of DEHP for 60 days. Data are expressed as mean ± SEM (*n* = 6), and values with different superscript letters (a denotes the highest value, followed by b, c, and d) significantly differ at the 0.05 level.

**Figure 5:**
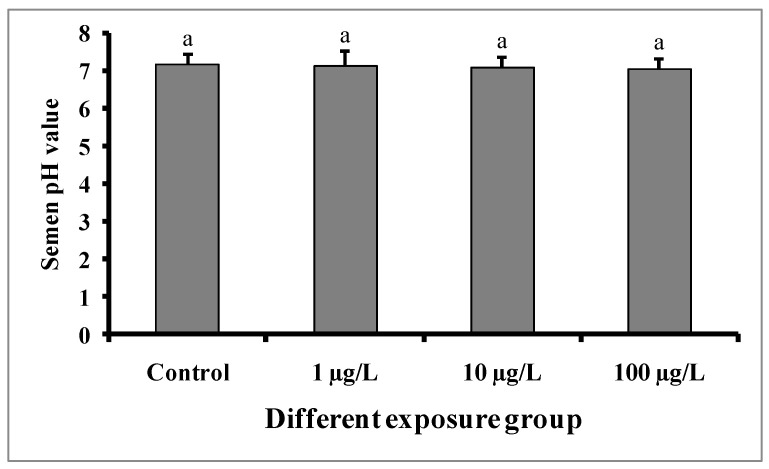
pH value in semen samples of male mature koi carp exposed to nominal 1, 10, and 100 µg/L of DEHP for 60 days. Data are expressed as mean ± SEM (*n* = 6), and values with superscript letters (a) do not significantly differ at the 0.05 level.

Semen volumes were significantly decreased with the increase in DEHP concentrations compared to the control group (Figure 4). The semen volume was 1.28 ± 0.02 mL in the control group, while 1.12 ± 0.01, 0.98 ± 0.02, and 0.84 ± 0.01 mL were recorded in the groups treated with 1, 10, and 100 µg/L of DEHP, respectively. There were no significant differences in the pH of semen samples from the treated groups as well as the control group (Figure 5).

### 3.4. Changes in Sperm Motility

**Figure 6:**
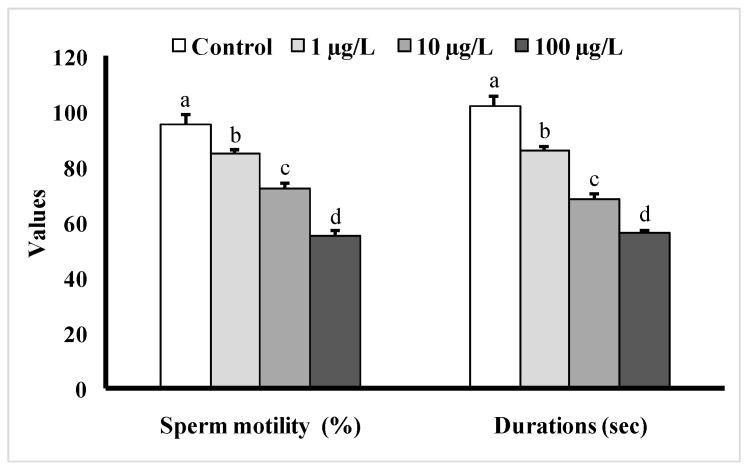
Sperm motility (%) as well as motility durations (s) of male mature koi carp exposed to nominal 1, 10, and 100 µg/L of DEHP for 60 days. Data are expressed as mean ± SEM (*n* = 6), and values with different superscript letters (a denotes the highest value, followed by b, c, and d) significantly differ at the 0.05 level.

Fish exposed to different doses of DEHP showed significant decreases in sperm motility as well as sperm motility durations as compared to the control group (Figure 6). The sperm motility was the lowest (55 ± 1.98%) in the group treated with 100 µg/L of DEHP, and the duration of sperm motility was found to be 56 ± 1.46 s, while in the control group, the motility value was found to be 95 ± 3.96%, and the motility duration was 102 ± 3.76 s.

### 3.5. Changes in Spermatocrit Value

**Figure 7:**
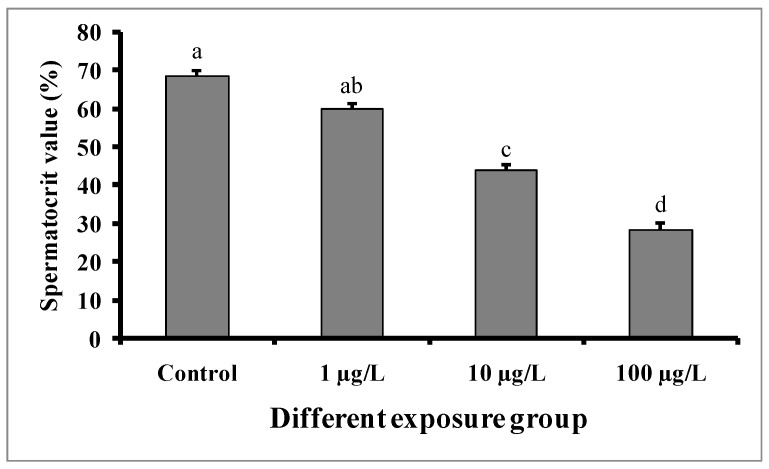
Spermatocrit values (%) of male mature koi carp exposed to nominal 1, 10, and 100 µg/L of DEHP for 60 days. Data are expressed as mean ± SEM (*n* = 6), and values with different superscript letters (a denotes the highest value, followed by b, c, and d) significantly differ at the 0.05 level.

Significant decreases in spermatocrit values were observed in the semen samples of males exposed to medium (10 µg/L) and higher concentrations (100 µg/L) of DEHP (Figure 7). The spermatocrit value was lowest (28.5 ± 1.52%) in the fish treated with a higher dose (100 µg/L) of DEHP, while the value was 68.5 ± 1.5% in the control group.

### 3.6. Changes in Sperm Density

**Figure 8:**
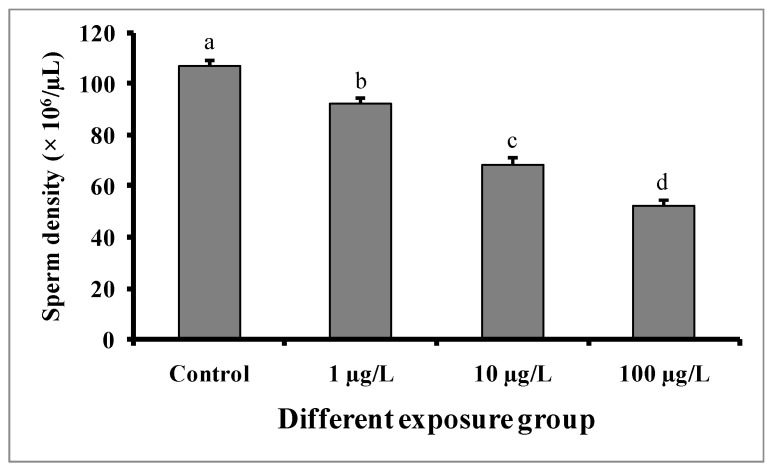
Sperm density (× 10^6^/µL) of male mature koi carp exposed to nominal 1, 10, and 100 µg/L of DEHP for 60 days. Data are expressed as mean ± SEM (*n* = 6), and values with different superscript letters (a denotes the highest value, followed by b, c, and d) significantly differ at the 0.05 level.

Compared to the control group, significant dose-dependent decreases in sperm density were observed in fishes in all DEHP-treated groups (Figure 8). In the control group, the sperm density was found to be 106.8 ± 2.08 × 10^6^/µL, while the lowest sperm density (52.6 ± 1.56 × 10^6^/µL) was recorded in the group treated with 100 µg/L of DEHP.

### 3.7. Changes in Biochemical Parameters of Seminal Fluid

The biochemical compositions of the seminal fluid are shown in Table 2. The glucose and cholesterol levels in the seminal fluid were significantly increased, while the total protein levels in the seminal fluid were significantly decreased in fishes treated with increasing concentrations of DEHP compared to the control group. The glucose and cholesterol levels in the seminal fluid of the control group were 6.4 ± 0.06 and 4.2 ± 0.04 mg/dL, respectively. However, in the group treated with 100 µg/L of DEHP, the glucose and cholesterol levels were found to be 12.8 ± 0.08 and 8.45 ± 0.06 mg/dL, respectively. In the case of total protein, the value was 0.86 ± 0.02 g/dL in the control group, while it was 0.38 ± 0.01 g/dL in the group treated with 100 µg/L of DEHP.

The ionic composition of the seminal fluid of DEHP-exposed fishes (all groups) showed significant decreases in Na and K ions. The Na and K ions in the seminal fluid of the control group were 122.2 ± 2.86 and 38.8 ± 1.24 mmol/L, respectively. However, in the group treated with 100 µg/L of DEHP, the Na and K ions were found to be 72.6 ± 1.98 and 22.4 ± 0.96 mmol/L, respectively. The Ca and Mg ions were not significantly different in the DEHP-treated groups, as well as in the control group.

### 3.8. Sex Steroid Levels

**Figure 9:**
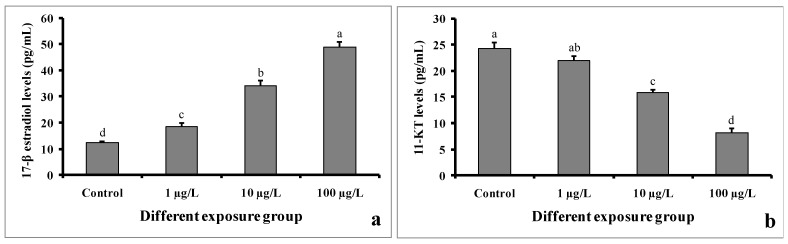
Sex steroid levels of 17-β estradiol (**a**) and 11-keto testosterone (**b**) (pg/mL) of male mature koi carp exposed to nominal 1, 10, and 100 µg/L of DEHP for 60 days. Data are expressed as mean ± SEM (*n* = 6), and values with different superscript letters (a denotes the highest value, followed by b, c, and d) significantly differ at the 0.05 level.

The levels of 17-β estradiol in serum samples were found to be higher in DEHP-treated males as compared to control males (Figure 9a). In the control group, the value was found to be 12.22 ± 0.5 pg/mL. However, the value was 48.86 ± 1.94 pg/mL in the group treated with 100 µg/L of DEHP.

In contrast, the 11-keto testosterone levels in the serum samples of DEHP-treated males were significantly decreased compared to those of the control males (Figure 9b). In the group treated with 100 µg/L of DEHP, the value was found to be 8.18 ± 0.75 pg/mL. However, the value was 24.26 ± 1.16 pg/mL in the control group.

### 3.9. Gene Expression

**Figure 10:**
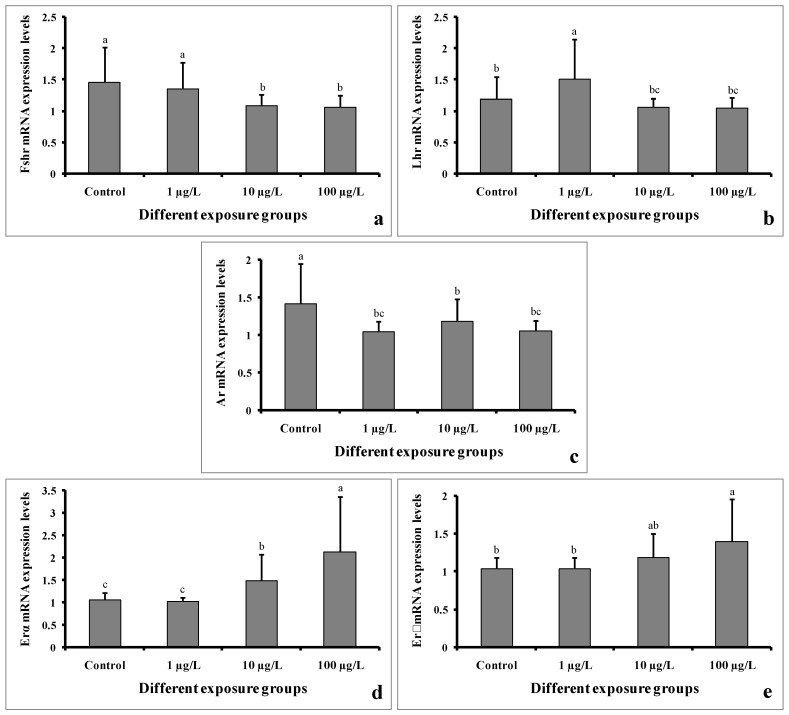
mRNA expression levels of Fshr (**a**), Lhr (**b**), Ar (**c**), Erα (**d**), and Erβ (**e**) of male mature koi carp exposed to nominal 1, 10, and 100 µg/L of DEHP for 60 days. Data are expressed as mean ± SEM (*n* = 6), and values with different superscript letters (a denotes the highest value, followed by b and c) significantly differ at the 0.05 level.

The mRNA expression levels of Fshr were significantly decreased in the groups treated with 10 and 100 µg/L of DEHP. However, the level remained unchanged in the group treated with 1 µg/L of DEHP compared to the control group (Figure 10a). The Lhr mRNA expression level was significantly increased in the group treated with 1 µg/L of DEHP. However, the levels were significantly decreased in the groups treated with 10 and 100 µg/L of DEHP (Figure 10b). Interestingly, the mRNA expression levels of Ar were significantly decreased in all of the treated groups compared to the control group (Figure 10c). On the contrary, the Erα mRNA expression levels were significantly increased in the groups treated with 10 and 100 µg/L of DEHP. However, in the group treated with 1 µg/L of DEHP, the levels showed no significant differences (Figure 10d). Similarly, the expression levels of Erβ mRNA were significantly increased in the groups treated with 10 and 100 µg/L of DEHP. However, the level did not significantly differ in the group treated with 1 µg/L of DEHP (Figure 10e).

## 4. Discussion

The aim of this study was to explore the effects on gamete quality parameters in male *C. carpio* after 60 days of exposure to environmentally relevant concentrations of DEHP. In this study, we found that 60 days of exposure to DEHP significantly decreased the GSI in males treated with 10 and 100 µg/L. Similarly, the GSI was decreased in male *D. rerio* exposed to 0.5 μg/L of DEHP for 180 days [37]. However, the GSI was not changed in DEHP-treated (1, 10, and 100 µg/L) male *C. auratus* after a 30-day exposure period [29]. In our study, the histological analysis of the testes revealed impaired histoarchitecture, including the presence of inflammatory cells, intratubular vacuoles, and swollen seminiferous tubules in DEHP-treated groups in a dose-dependent manner. Similarly, the 14 days of DEHP exposure (100 and 400 µg/L) to *C. gariepinus* displayed the distortion and degeneration of the tubular epithelium and seminiferous tubule, intertubular vacuolation, the condensation of tubular cells and inflammation in testes [31]. However, the histological analysis of 90 days of DEHP exposure (100 µg/L) in *D. rerio* identified a significant decline in spermatozoa content [30]. The accumulation of DEHP in tissue samples of exposed fishes has been confirmed in the present study using GC-MS technology. Excluding the control group, DEHP was detected in each group. A higher level of DEHP was detected in the higher treatment group, i.e., 100 µg/L, and in control group, DEHP was below the limit of detection. Similarly, a higher level of DEHP was detected in the liver tissue of *C. gariepinus* at a higher dose of DEHP exposure (400 µg/L) for 14 days [31]. In our study, the accumulation of DEHP in the testes tissue of exposed fishes resulted in changes in severe damage to the seminiferous tubules.

In the present study, sperm production was decreased in all of the DEHP-treated groups, which is consistent with earlier studies on *C. auratus* [29]. However, the pH of semen samples from the DEHP-treated fishes was not significantly different from that of the control group. The observed decrease in sperm production might be due to DEHP interfering with spermatogenesis [15,27]. Sperm motility is an important indicator of sperm quality and is usually expressed as the percentage and duration of motile sperm after activation. In our study, the sperm motility as well as the duration of sperm motility were significantly decreased in each of the DEHP-treated groups compared to the control group. Similar results were previously reported in DEHP-exposed *C. auratus* [29]. The determination of sperm density has been traditionally used to assess sperm quantity and is one characteristic that may influence the fertilization rate in fish via biochemical and enzymatic activities in the seminal fluid [38]. The present study revealed significant decreases in spermatocrit values of *C. carpio* exposed to 10 and 100 µg/L of DEHP. The fish in DEHP-exposed groups also showed significant dose-dependent decreases in sperm density. Similarly, sperm density and spermatocrit values were decreased in *C. auratus* treated with 10 and 100 µg/L of DEHP [29].

Fish seminal fluid is characterized by a low protein concentration; substantial mineral compounds like sodium (Na), potassium (K), calcium (Ca), and magnesium (Mg); and low concentrations of organic substances (glucose, cholesterol, and others). It plays a crucial physio-endocrinological role in supporting spermatozoa after the release of sperm from the testes into the sperm duct and subsequently after the discharge of sperm into the aquatic environment [32]. In the present study, the seminal glucose and cholesterol levels were significantly increased, whereas the total protein levels were significantly decreased in DEHP-treated fishes. On the contrary, the Na and K ions were lower in the fish in DEHP-treated groups. However, the other ionic parameters like Ca and Mg did not change in DEHP-exposed groups as well as in the control group. No such work has been carried out so far on this aspect in the case of fish, and to our knowledge, this is the first study suggesting that DEHP reduced sperm quality, leading to alterations in biochemical properties of seminal fluid.

To better understand how DEHP reduced sperm production, we measured 11-keto testosterone levels, which regulate fish spermatogenesis [39,40]. 11-keto testosterone levels were significantly decreased in DEHP-treated *C. carpio* following 60 days of exposure, which is consistent with another study where 30 days of exposure of DEHP decreased 11-keto testosterone levels in mature male *C. auratus* [29]. This study suggested that DEHP is capable of inhibiting 11-keto testosterone production in male fish following chronic exposure and also revealed that DEHP reduced sperm production due to the inhibition of 11-keto testosterone production. However, we found significant increases in 17-β estradiol levels in DEHP-treated *C. carpio* after the exposure period, which suggests that DEHP causes estrogenic activity in males. Similarly, the estrogenic effect was also found in male *O. melastigma* after 180 days of exposure to 0.1 and 0.5 mg/L of DEHP [28].

In the present study, we analyzed the transcript profiles of five genes (Fshr, Lhr, Ar, Erα, and Erβ) involved in sexual function and reproductive success in teleosts. The results of our study showed that Fshr and Lhr were significantly decreased in groups treated with 10 and 100 µg/L. However, the mRNA expression levels of Ar were decreased in all of the DEHP-treated groups. Nearly similar results were reported previously in *C. auratus* [29]. On the contrary, the mRNA expression levels of Erα and Erβ were significantly increased in the groups treated with 10 and 100 µg/L of DEHP, suggesting an estrogenic effect in males. However, the injection of 0.5–5000 mg/kg of DEHP did not change the mRNA expression levels of Erα and Erβ in *D. rerio* [15].

The present study provides novel information on the emerging environmental pollutant DEHP on reproductive disorders in koi carp (*C. carpio*). DEHP can reduce sperm production, density, motility, and spermatocrit value in exposed fishes following 60 days of exposure. Significant decreases in 11-keto testosterone and significant increases in 17-β estradiol levels were observed after the exposure period. The mRNA expression levels of reproduction-related genes were altered in a dose-dependent manner. The results of this study proved that environmentally relevant exposure to DEHP may be a factor in male fish gamete quality reduction, which may be relevant for further molecular studies on other animals.

## Figures and Tables

**Table 1:** Primer sets used in amplification as well as qPCR for mRNA expression study of reproduction-related genes.

Target Gene	Oligo Sequence	Annealing Temperature	Product Size	Source
Fshr	F: 5′-CACACCAGATGCATTCAACC-3′ R: 5′-GTCTGCAAATGCCAGGTGGC-3′	58 °C	180 bp	This study
Lhr	F: 5′-GCCATGCCTTCAATGGAAC-3′ R: 5′-CAGCATCAGCACAGACTCCAG-3′	56 °C	172 bp	This study
Ar	F: 5′-GGGCCAAAGGACTTCCAGG-3′ R: 5′-GCTTCATCTGAACACAGTGC-3′	56 °C	212 bp	This study
Erα	F: 5′-CTTTGCTCAGGATCTCATCC-3′ R: 5′-CACCATGAAGCTGTCCATC-3′	55 °C	180 bp	This study
Erβ	F: 5′-GGAGTGCTGCTGGTTAGAGG-3′ R: 5′-CTTCAGTTCTCTGAACCTGG-3′	54 °C	216 bp	This study
18S rRNA	F: 5′-GAGTATGGTTGCAAAGCTGAAAC-3′ R: 5′-AATCTGTCAATCCTTTCCGTGTCC-3′	56 °C	128 bp	[36]

**Table 2:** Changes in biochemical parameters of seminal fluid in control and different DEHP-exposed fishes. Data are expressed as mean ± SEM (*n* = 6), and values with different superscript letters (a denotes the highest value, followed by b, c, and d) significantly differ at the 0.05 level.

Components	Control	1 µg/L DEHP	10 µg/L DEHP	100 µg/L DEHP
Glucose (mg/dL)	6.4 ± 0.06 ^d^	8.6 ± 0.08 ^c^	10.45 ± 0.04 ^b^	12.8 ± 0.08 ^a^
Cholesterol (mg/dL)	4.2 ± 0.04 ^c^	4.85 ± 0.08 ^c^	6.6 ± 0.06 ^b^	8.45 ± 0.06 ^a^
Total protein (g/dL)	0.86 ± 0.02 ^a^	0.78 ± 0.02 ^ab^	0.56 ± 0.01 ^b^	0.38 ± 0.01 ^c^
Sodium (mmol/L)	122.2 ± 2.86 ^a^	116.6 ± 1.88 ^a^	92.45 ± 1.40 ^b^	72.6 ± 1.98 ^c^
Potassium (mmol/L)	38.8 ± 1.24 ^a^	34.65 ± 0.88 ^ab^	30.2 ± 1.08 ^b^	22.4 ± 0.96 ^c^
Calcium (mmol/L)	0.66 ± 0.01 ^a^	0.65 ± 0.02 ^a^	0.64 ± 0.02 ^a^	0.62 ± 0.04 ^a^
Magnesium (mmol/L)	1.18 ± 0.12 ^a^	1.16 ± 0.08 ^a^	1.15 ± 0.06 ^a^	1.14 ± 0.04 ^a^

## Data Availability

Data supporting the reported results are contained within the article.

## Supplementary Materials

The following supporting information can be downloaded at: https://www.mdpi.com/article/10.3390/cimb45090467/s1. Section S1: Sample preparation and GCMS conditions; Section S2: Primer designing, amplification, and sequencing; Figure S1: Agarose gel electrophoresis of PCR products of the 18S rRNA (a), Fshr genes (b), Lhr genes (c), Ar genes (d), Erα genes (e), and Erβ genes (f) with 100 bp marker (M), cDNA of control male (1), cDNA of male treated with 1 µg/L (2), cDNA of male treated with 10 µg/L (3), and cDNA of male treated with 100 µg/L (4).

## References

[1] Bold J., Swinburne D. (2022). Pre-conceptual guidelines for men: A review of male infertility experience, including nutrition and lifestyle factors. Dietetics.

[2] Kumar N., Singh A.K. (2022). Impact of environmental factors on human semen quality and male fertility: A narrative review. Environ. Sci. Eur..

[3] Calogero A.E., Fiore M., Giacone F., Altomare M., Asero P., Ledda C., Romeo G., Mongioì L.M., Copat C., Giuffrida M. (2021). Exposure to multiple metals/metalloids and human semen quality: A cross-sectional study. Ecotoxicol. Environ. Saf..

[4] Virtanen H.E., Jørgensen N., Toppari J. (2017). Semen quality in the 21st century. Nat. Rev. Urol..

[5] Montano L., Giorgini E., Notarstefano V., Notari T., Ricciardi M., Piscopo M., Motta O. (2023). Raman Microspectroscopy evidence of microplastics in human semen. Sci. Total Environ..

[6] Yang W.K., Chiang L.F., Tan S.W., Chen P.J. (2018). Environmentally relevant concentrations of di-(2-ethylhexyl) phthalate exposure alter larval growth and locomotion in medaka fish via multiple pathways. Sci. Total Environ..

[7] Kastner J., Cooper D.G., Marić M., Dodd P., Yargeau V. (2012). Aqueous leaching of di-(2-ethylhexyl) phthalate and “green” plasticizers from poly (vinyl chloride). Sci. Total Environ..

[8] Magdouli S., Daghrir R., Brar S.K., Drogui P., Tyagi R.D. (2013). Di-(2-ethylhexyl) phtalate in the aquatic and terrestrial environment: A critical review. J. Environ. Manag..

[9] Bergé A., Cladière M., Gasperi J., Coursimault A., Tassin B., Moilleron R. (2013). Meta-analysis of environmental contamination by phthalates. Environ. Sci. Pollut. Res..

[10] Martine B., Cendrine D., Fabrice A., Marc C. (2013). Assessment of adult human exposure to phthalate esters in the urban centre of Paris (France). Bull. Environ. Contam. Toxicol..

[11] Schettler T.E.D. (2006). Human exposure to phthalates via consumer products. Int. J. Androl..

[12] Rowdhwal S.S.S., Chen J. (2018). Toxic effects of di-(2-ethylhexyl) phthalate: An overview. Biomed Res. Int..

[13] Yuwatini E., Hata N., Taguchi S. (2006). Behavior of di-(2-ethylhexyl) phthalate discharged from domestic waste water into aquatic environment. J. Environ. Monit..

[14] Khan M.H., Jung J.Y. (2008). Ozonation catalyzed by homogeneous and heterogeneous catalysts for degradation of DEHP in aqueous phase. Chemosphere.

[15] Uren-Webster T.M., Lewis C., Filby A.L., Paull G.C., Santos E.M. (2010). Mechanisms of toxicity of di-(2-ethylhexyl) phthalate on the reproductive health of male zebrafish. Aquat. Toxicol..

[16] Kalo D., Roth Z. (2017). Low level of mono-(2-ethylhexyl) phthalate reduces oocyte developmental competence in association with impaired gene expression. Toxicology.

[17] Bhattacharya N., Dufour J.M., Vo M.N., Okita J., Okita R., Kim K.H. (2005). Differential effects of phthalates on the testes and the liver. Biol. Reprod..

[18] Corton J.C., Lapinskas P.J. (2005). Peroxisome proliferator-activated receptors: Mediators of phthalate ester-induced effects in the male reproductive tract. Toxicol. Sci..

[19] Grande S.W., Andrade A.J., Talsness C.E., Grote K., Chahoud I. (2006). A dose-response study following in utero and lactational exposure to di-(2-ethylhexyl) phthalate: Effects on female rat reproductive development. Toxicol. Sci..

[20] Howdeshell K.L., Wilson V.S., Furr J., Lambright C.R., Rider C.V., Blystone C.R., Hotchkiss A.K., Gray L.E. (2008). A mixture of five phthalate esters inhibits fetal testicular testosterone production in the sprague-dawley rat in a cumulative, dose-additive manner. Toxicol. Sci..

[21] Lee Y.J., Lee E., Kim T.H., Choi J.S., Lee J., Jung K.K., Kwack S.J., Kim K.B., Kang T.S., Han S.Y. (2009). Effects of di-(2-ethylhexyl) phthalate on regulation of steroidogenesis or spermatogenesis in testes of Sprague-Dawley rats. J. Health Sci..

[22] Herr C., Nieden A.Z., Koch H.M., Schuppe H.C., Fieber C., Angerer J., Eikmann T., Stilianakis N.I. (2009). Urinary di-(2-ethylhexyl) phthalate (DEHP)—Metabolites and male human markers of reproductive function. Int. J. Hyg. Environ. Health.

[23] Mendiola J., Jørgensen N., Andersson A.M., Calafat A.M., Silva M.J., Redmon J.B., Sparks A., Drobnis E.Z., Wang C., Liu F. (2011). Associations between urinary metabolites of di-(2-ethylhexyl) phthalate and reproductive hormones in fertile men. Int. J. Androl..

[24] Mendiola J., Meeker J.D., Jørgensen N., Andersson A.M., Liu F., Calafat A.M., Redmon J.B., Drobnis E.Z., Sparks A.E., Wang C. (2012). Urinary concentrations of di-(2-ethylhexyl) phthalate metabolites and serum reproductive hormones: Pooled analysis of fertile and infertile men. J. Androl..

[25] Specht I.O., Toft G., Hougaard K.S., Lindh C.H., Lenters V., Jönsson B.A., Heederik D., Giwercman A., Bonde J.P.E. (2014). Associations between serum phthalates and biomarkers of reproductive function in 589 adult men. Environ. Int..

[26] Wang Y.X., You L., Zeng Q., Sun Y., Huang Y.H., Wang C., Wang P., Cao W.C., Yang P., Li Y.F. (2015). Phthalate exposure and human semen quality: Results from an infertility clinic in China. Environ. Res..

[27] Corradetti B., Stronati A., Tosti L., Manicardi G., Carnevali O., Bizzaro D. (2013). Bis-(2-ethylexhyl) phthalate impairs spermatogenesis in zebrafish (*Danio rerio*). Reprod. Biol..

[28] Ye T., Kang M., Huang Q., Fang C., Chen Y., Shen H., Dong S. (2014). Exposure to DEHP and MEHP from hatching to adulthood causes reproductive dysfunction and endocrine disruption in marine medaka (*Oryzias melastigma*). Aquat. Toxicol..

[29] Golshan M., Hatef A., Socha M., Milla S., Butts I.A., Carnevali O., Rodina M., Sokołowska-Mikołajczyk M., Fontaine P., Linhart O. (2015). Di-(2-ethylhexyl)-phthalate disrupts pituitary and testicular hormonal functions to reduce sperm quality in mature goldfish. Aquat. Toxicol..

[30] Ma Y.B., Jia P.P., Junaid M., Yang L., Lu C.J., Pei D.S. (2018). Reproductive effects linked to DNA methylation in male zebrafish chronically exposed to environmentally relevant concentrations of di-(2-ethylhexyl) phthalate. Environ. Pollut..

[31] Adeogun A.O., Ibor O.R., Imiuwa M.E., Omogbemi E.D., Chukwuka A.V., Omiwole R.A., Arukwe A. (2018). Endocrine disruptor responses in African sharptooth catfish (*Clarias gariepinus*) exposed to di-(2-ethylhexyl)-phthalate. Comp. Biochem. Physiol. C Toxicol. Pharmacol..

[32] Alavi S.M.H., Cosson J. (2006). Sperm motility in fishes. (II) Effects of ions and osmolality: A review. Cell Biol. Int..

[33] Linhart O., Alavi S.M.H., Rodina M., Gela D., Cosson J. (2008). Comparison of sperm velocity, motility and fertilizing ability between firstly and secondly activated spermatozoa of common carp (*Cyprinus carpio*). J. Appl. Ichthyol..

[34] Alavi S.M.H., Cosson J., Kazemi R. (2006). Semen characteristics in *Acipenser persicus* in relation to sequential stripping. J. Appl. Ichthyol..

[35] Rosengrave P., Taylor H., Montgomerie R., Metcalf V., Mc-Bride K., Gemmell N.J. (2009). Chemical composition of seminal and ovarian fluids of chinook salmon (*Oncorhynchus tshawytscha*) and their effects on sperm motility traits. Comp. Biochem. Physiol. Part A Mol. Integr. Physiol..

[36] Tang Y.K., Yu J.H., Xu P., Li J.L., Li H.X., Ren H.T. (2012). Identification of housekeeping genes suitable for gene expression analysis in Jian carp (*Cyprinus carpio* var. jian). Fish Shellfish Immunol..

[37] Muhammad S., Zhang Z., Pavase T.R., Guo H. (2018). Long-term exposure of two plasticizers di-(2-ethylhexyl)-phthalate (DEHP) and acetyl tributyl citrate (ATBC): Toxic effects on gonadal development and reproduction of zebrafish (*Danio rerio*). Indian J. Geo-Mar. Sci..

[38] Rurangwa E., Kime D.E., Ollevier F., Nash J.P. (2004). The measurement of sperm motility and factors affecting sperm quality in cultured fish. Aquaculture.

[39] Miura T., Miura C.I. (2003). Molecular control mechanisms of fish spermatogenesis. Fish Physiol. Biochem..

[40] Ismail M.F.S., Siraj S.S., Daud S.K., Harmin S.A. (2011). Association of annual hormonal profile with gonad maturity of mahseer (*Tor tambroides*) in captivity. Gen. Comp. Endocrinol..

