# Supplementary material for: Effects of Di-(2-Ethylhexyl) Phthalate (DEHP) on Gamete Quality Parameters of Male Koi Carp (Cyprinus carpio)
Source: Curr Issues Mol Biol. 2023 Sep 11;45(9):7388–403. doi: 10.3390/cimb45090467 (PMC10529456; doi:10.3390/cimb45090467)
Supplement: Supplementary file 1 [file cimb-45-00467-s001.zip › cimb-2587604-supplementary.pdf]

Effects of di-(2-ethylhexyl) phthalate (DEHP) on gamete quality parameters of male koi carp (*Cyprinus carpio*)

Section S1 (Sample preparation and GCMS conditions)

The tissue samples (liver and testes) were homogenised in a glass homogenizer after being rinsed twice with 10 mL of deionized water. Then, as an internal standard, 0.04 µg of DEHP-D4 was added to all samples. To separate the organic layers, the homogenate was centrifuged (Hermle Z 323 K, Labour Technik, Beckum, Nordrhein-Westfalen, Germany) at 1000 × g for 10 min at 4 ºC with dichloromethane and hexane in a 1:1 (v/v) ratio for 10 min in an ultrasonic water bath. After removing the organic layer, the extraction was carried out twice more using the new solvent. The tissue extracts were evaporated under a mild nitrogen stream, the residue was reconstituted in 0.2 mL of hexane, and the supernatant was taken out and placed into an autosampler (AS 1310 Series Autosampler, Thermo Scientific, Waltham, MA, USA). Sample injection (1 µL) was performed in splitless mode with an inlet temperature of 300 ºC, while the column temperature was initially held at 200 ºC for 1 min and then increased at 30 ºC/min to 300 ºC. The carrier gas was nitrogen with a flow rate of 1.2 mL/min. Ions monitored for DEHP and DEHP-D4 were 149 and 153, respectively. All samples were analyzed in triplicate.

To avoid contamination, no plastic product was used, and each glassware was sequentially washed with 5% glassware cleaning agent (Borosil Glass Works Ltd., Mumbai, Maharastra, India) and rinsed with 50% hydrochloric acid and demineralized water. After then, they were air dried, covered with aluminium foil, and sterilized in a hot-air oven (BST-HAO-1125, Bionics Scientific, Sindhora Kalan, Delhi, India) at 200 ºC for 4 hrs.

Section S2 (Primer designing, amplification, and sequencing)

To date, no primer details are available to amplify the follicle-stimulating hormone receptor (Fshr), luteinizing hormone receptor (Lhr), androgen receptor (Ar), estrogen receptor alpha (Erα), and estrogen receptor beta (Erβ) of koi carp (*C. carpio*). So, the mRNA sequences of the above-mentioned gene of all closely related species (mainly Cypriniformes) were retrieved from the NCBI Gen-Bank (https://www.ncbi.nlm.nih.gov/genbank/). Then, these sequences were aligned, and the forward and reverse primers were designed with predicted amplification product sizes of 120-220 bp in length using MEGA-X software (https://www.megasoftware.net/). PCR

was performed on a Thermal Cycler Gene Amp PCR system 9700 (Applied Biosystems, Foster City, CA, USA). Briefly, 50 µL of the PCR reaction mixture consisted of 5 µL of 10× PCR buffer, 1 µL of 50 mM $MgCl_2$, 1 µL of 10 mM dNTP, 1 µL of 10 pmol of each primer, 100 ng of cDNA and 1 U Taq DNA polymerase. The thermal conditions consisted of initial denaturation (95 ºC for 2 min), 35 cycles of denaturation (94 ºC for 30 sec), annealing (Fshr: 58 ºC, Lhr and Ar: 56 ºC, Erα: 55 ºC and Erβ: 54 ºC for 1min), extension (72 ºC for 1.50 min) and final extension (72 ºC for 7 min) and the reaction was ended at 4 ºC. PCR products were separated on 2% agarose by ethidium bromide gel electrophoresis (Figure S1). The amplified genes were sequenced in forward and reverse directions using an ABI 3730XL capillary sequencer (Applied Biosystem, Foster City, CA, USA). Contigs were prepared by aligning forward and reverse sequences using DNA baser 7.0 (https://www.dnabaser.com/). The sequences of the above-mentioned genes were also subjected to BLAST-N search (https://blast.ncbi.nlm.nih.gov/Blast.cgi), and the results showed that the sequences have the highest similarity with their corresponding gene sequences of *C. carpio*.

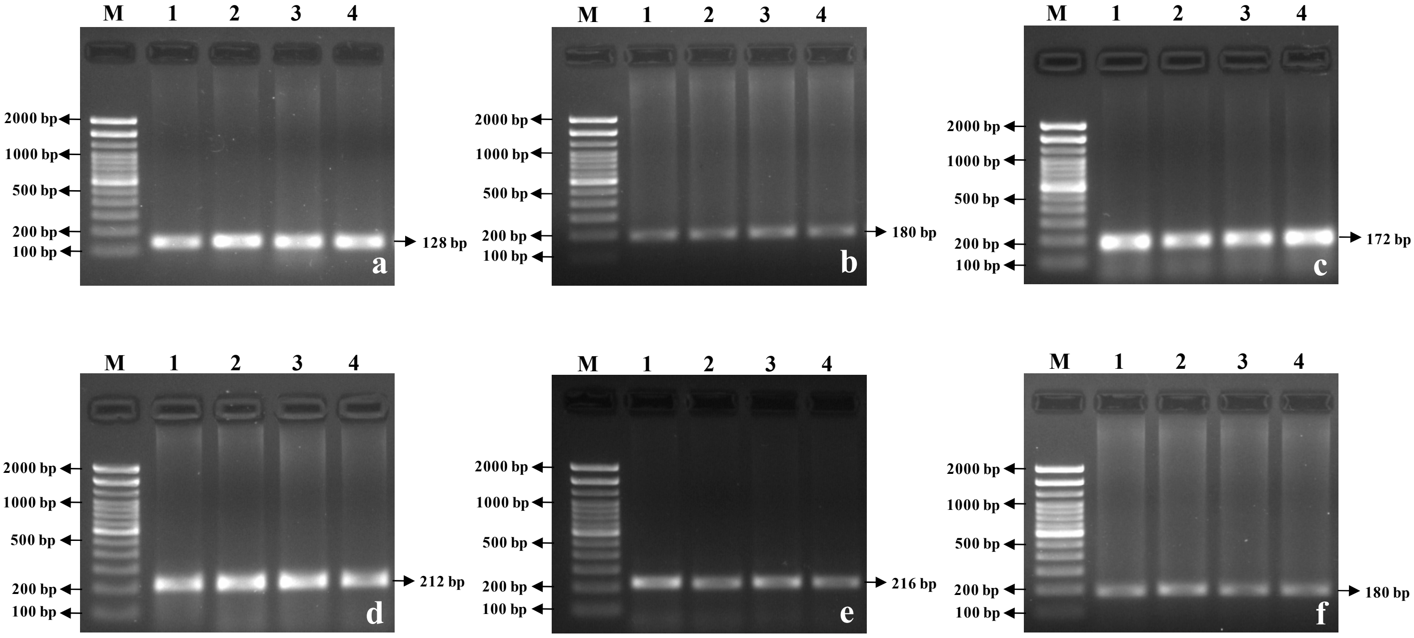

Figure S1. Agarose gel electrophoresis of PCR products of the 18S rRNA genes (a), Fshr genes (b), Lhr genes (c), Ar genes (d), Erα genes (e), and Erβ genes (f) with 100 bp marker (M), cDNA of control male (1), cDNA of 1 µg/L treated male (2), cDNA of 10 µg/L treated male (3), and cDNA of 100 µg/L treated male (4).
